# Impact of neutrophil-to-lymphocyte ratio throughout the course of chemoradiotherapy on overall survival and distant failure in unresectable stage III non-small cell lung cancer

**DOI:** 10.1007/s11604-021-01129-1

**Published:** 2021-05-17

**Authors:** Hiromitsu Kanzaki, Yasushi Hamamoto, Kei Nagasaki, Toshiyuki Kozuki

**Affiliations:** 1 Department of Radiation Oncology, National Hospital Organization Shikoku Cancer Center, Kou-160, Minami-umenomoto-machi, Matsuyama, Ehime 791-0280 Japan; 2 Department of Thoracic Oncology and Medicine, National Hospital Organization Shikoku Cancer Center, Kou-160, Minami-umenomoto-machi, Matsuyama, Ehime 791-0280 Japan

**Keywords:** Non-small cell lung cancer, Radiotherapy, Stage III, Neutrophil-to-lymphocyte ratio

## Abstract

**Purpose:**

Neutrophil-to-lymphocyte ratio (NLR) has been reported to be associated with treatment outcomes in various cancers; however, the optimal timing to measure NLR is unclear. In this study, “average-NLR” was newly devised, which reflects the NLR throughout the course of radiotherapy, and its usefulness was assessed for stage III non-small cell lung cancer (NSCLC) patients treated with chemoradiotherapy.

**Materials and methods:**

A total of 111 patients who received definitive chemoradiotherapy for unresectable stage III NSCLC were reviewed. Patient/tumor-related factors, treatment-related, and NLR-related factors (average-NLR, pre- and post-radiotherapy NLR, NLR-nadir, NLR-maximum) were assessed using univariate and multivariate analyses.

**Results:**

The median follow-up period was 43.8 months among the survivors. In the multivariate analysis, average-NLR and post-radiotherapy NLR were significant factors for the overall survival (OS) (*p* = 0.016 and 0.028) and distant failure (DF) (*p* = 0.008 and 0.040). For the patients with low, intermediate, and high average-NLR, the median OS was 41.2, 37.7, and 14.8 months, respectively, and the median DF free time was 52.5, 13.5, and 8.9 months, respectively.

**Conclusion:**

Average-NLR and post-radiotherapy NLR were significant factors for the OS and DF. Average-NLR, which was available immediately after the completion of chemoradiotherapy, seemed to be helpful for treatment decisions.

## Introduction

Non-small cell lung cancer (NSCLC) is one of the most common and fatal cancer worldwide as of 2018 [1]. Platinum-based concurrent chemoradiotherapy has been the standard therapy for the management of unresectable stage III NSCLC. Recently, the PACIFIC trial showed that the addition of consolidation immune checkpoint inhibitors to chemoradiotherapy (CRT) had prolonged survival in patients with unresectable stage III NSCLC [2]. This trial suggested that the immune system plays an important role in tumor control of NSCLC treated with CRT.

In addition, inflammation impacts every step of tumorigenesis, including initiation, tumor promotion, and metastatic progression [3]. The inflammatory response plays a major role in tumor progression through the tumor microenvironment [3–5]. Various types of immune and inflammatory cells are frequently observed in tumors. Immune cells produce cytokines, chemokines, growth factors, prostaglandins, and reactive oxygen and nitrogen species, and these factors affect malignant tumor cells [3]. Therefore, both immune system and inflammation potentially have large impact on tumor progression and treatment outcomes. The inflammatory response often leads to an increased neutrophil count. Lymphopenia often reflects the deterioration of the immune response and often reduces survival in patients with different solid tumors [6, 7]. Therefore, an increase in the neutrophil-to-lymphocyte ratio (NLR) in peripheral blood may reflect two contexts: an increase in the inflammatory response and deterioration of anti-tumor immune response. A meta-analysis showed that elevated NLR was associated with decreased survival in patients with solid tumors [8]. Some studies also reported that NLR measured before radiotherapy, during radiotherapy, at the completion of radiotherapy, and several months after radiotherapy was associated with treatment outcomes of NSCLC treated with definitive or palliative radiotherapy [9–13]. However, there had been no consensus on the best timing to measure NLR and which of the best NLR-related factors have been reported. Measuring NLR at a certain time point can be influenced by incidental events, such as temporary infection. NLR-related factors available early after the completion of CRT, that is, before the start of consolidation immune checkpoint inhibitors, might be clinically useful.

Therefore, in this study, “average-NLR” was newly devised as a method for evaluating the influence of NLR throughout the CRT period. The usefulness of average-NLR for predicting treatment outcomes in patients with NSCLC treated with concurrent CRT was assessed.

## Materials and methods

### Patients

Between January 2010 and October 2018, a total of 149 consecutive unresectable stage III NSCLC patients treated with definitive concurrent CRT at a single institution were reviewed. This observational, retrospective, cohort study design was approved by the institutional ethics review board. Thirty-eight patients were excluded for the following reasons: (1) discontinuation of radiotherapy (*n* = 14); (2) double cancer and/or history of cancer treatment within the last 5 years (*n* = 15); (3) salvage surgery after radiotherapy (*n* = 2); (4) radiotherapy period exceeding 60 days (*n* = 4); and (5) details of combination therapy were unknown (*n* = 3). The remaining 111 patients were reviewed in this study. For tumor staging, fluorodeoxyglucose positron emission tomography/computed tomography was performed on 99 patients (89.2%), contrast-enhanced computed tomography (CE-CT) on 12 patients (10.8%), brain contrast-enhanced magnetic resonance imaging (CE-MRI) on 87 patients (78.4%), and brain CE-CT on 21 patients (18.9%). Three patients (2.7%) underwent no imaging studies for brain metastases. Stages of NSCLC were based on the Union for International Cancer Control, TNM Classification, seventh edition. Forty-nine (44.1%) and 62 patients (55.9%) had stage IIIA and IIIB NSCLC, respectively. Surveillance computed tomography imaging was performed every 3–6 months within 3 years after radiotherapy and, thereafter, every 6–12 months. Follow-up brain CE-MRI scans were not routinely performed for all patients unless there were suspicious symptoms of brain metastases during the follow-up. Three of the 111 patients (2.7%) were excluded from the analysis of failures because their post-radiotherapy images were not available.

### Radiotherapy and combination chemotherapy

Patients were treated with three-dimensional conformal radiotherapy. Radiotherapy was delivered using 4–10 MV photons with a linear accelerator (Clinac 21-EX or TrueBeam, Varian Medical Systems, Palo Alto, California, United States). All patients received 60 Gy of radiation therapy in 30 fractions. In principle, lymph node stations adjacent to gross tumor volumes were included in the irradiation fields as elective nodal irradiation. The regimens of concurrent chemotherapy are presented in Table 1. After CRT, 41 patients (36.9%) underwent consolidation of cytotoxic anticancer agents for less than 2 months after CRT. None of the patients underwent consolidation therapy using immune checkpoint inhibitors, and induction chemotherapy before CRT.

**Table 1:** Characteristics of unresectable stage III NSCLC patients

Age (years)	Median 65	(Range, 33–88)	
Sex	Male	89	(80.2%)
	Female	22	(19.8%)
ECOG-PS	0	40	(36.0%)
	1	63	(56.8%)
	2	7	(6.3%)
	3	1	(0.9%)
Histology	SCC	48	(43.2%)
	ADC	55	(49.5%)
	LCNEC	1	(0.9%)
	Pleomorphic	1	(0.9%)
	NSCLC, Unspecified	6	(5.4%)
Stage UICC-TNM 7th	IIIA	49	(44.1%)
	IIIB	62	(55.9%)
Combined chemotherapy	CDDP + DOC	55	(49.5%)
	CBDCA + PTX	24	(21.6%)
	CDDP + VNR	17	(15.3%)
	Other Platinum doublet	6	(5.4%)
	Low-dose CBDCA	8	(7.2%)
	Pemetrexed	1	(0.9%)
Consolidation chemotherapy	Yes	41	(36.9%)
	No	70	(63.1%)
Treatment period (days)	Median 46	(Range, 40–57)	
NLR-pre-RT	Median 3.1	(Range, 1.0–11.3)	
NLR-nadir	Median 2.3	(Range, 0.1–7.9)	
NLR-maximum	Median 14.7	(Range, 4.5–197.5)	
NLR-post-RT	Median 4.2	(Range, 1.6–48.4)	
Average-NLR	Median 6.5	(Range, 2.5–36.6)	

*ECOG-PS* Eastern Cooperative Oncology Group performance status, *UICC-TNM* Union for International Cancer Control TNM Classification, *NLR-pre-RT* neutrophil-to-lymphocyte ratio within 2 weeks before radiotherapy, *NLR-nadir* the minimal neutrophil-to-lymphocyte ratio in blood sampling during radiotherapy, *NLR-maximum* the maximal neutrophil-to-lymphocyte ratio in blood sampling during radiotherapy, *NLR-post-RT* neutrophil-to-lymphocyte ratio measured in 3–4 months after radiotherapy, *Average-NLR* the area under the line graph of NLR counts during radiotherapy was divided by days of treatment period, *SCC* squamous cell carcinoma, *ADC* adenocarcinoma, *LCNEC* large cell neuroendocrine carcinoma, *NSCLC* non-small cell lung cancer, *CDDP* cisplatin, *DOC* docetaxel, *CBDCA* carboplatin, *PTX* paclitaxel, *VNR* vinorelbine

### Neutrophil-to-lymphocyte ratio

To evaluate the influence of inflammatory and immune response throughout radiotherapy on treatment outcomes and to reduce the effect of the treatment period, an average-NLR was devised. NLR was calculated as the absolute neutrophil count divided by the absolute lymphocyte count in the blood sample. Average-NLR was defined as the area under the line graph of NLR counts during radiotherapy was divided by days of treatment period. Blood sampling for NLR at the start of radiotherapy was performed within 2 weeks before radiotherapy. For the blood sampling for NLR at the end of radiotherapy, the last blood sampling during radiotherapy was used. In principle, blood samplings were performed every week during radiotherapy. Blood sampling was performed 4–14 times (median, 8 times) during radiotherapy in each patient. In 105 patients (94.6%), at least seven times blood sampling data were used to calculate average-NLR. NLR-pre-RT was defined as the NLR within 2 weeks before radiotherapy. NLR-nadir was defined as the minimal NLR during radiotherapy. NLR-maximum was defined as the maximal NLR during radiotherapy. NLR-post-RT was defined as the NLR at 3–4 months after the completion of radiotherapy.

The median of NLR-pre-RT was 3.1 (first quarter (1Q), 1.0–2.3; second quarter (2Q), 2.3–3.1; third quarter (3Q), 3.1–4.1; and fourth quarter (4Q), 4.1–11.3). The median of average-NLR was 6.5 (1Q, 2.5–4.9; 2Q, 4.9–6.5; 3Q, 6.5–9.2; and 4Q, 9.2–36.6). The median of NLR-nadir was 2.3 (1Q, 0.1–1.5; 2Q, 1.5–2.3; 3Q, 2.3–3.2; and 4Q, 3.2–7.9). The median of NLR-maximum was 14.7 (1Q, 4.5–8.7; 2Q, 8.7–14.7; 3Q, 14.7–23.0; and 4Q, 23.0–197.5). The median of NLR-post-RT was 4.2 (1Q, 1.6–3.0; 2Q, 3.0–4.2; 3Q, 4.2–6.5; and 4Q, 6.5–48.4), and NLR-post-RT was not available for eight patients. We classified each NLR factor as follows to clarify the effect on treatment outcome: low NLR (1Q), intermediate NLR (2Q and 3Q), and high NLR (4Q). The ranges of NLR-pre-RT were divided into low (1.0–2.3), intermediate (2.3–4.1), and high (4.1–11.3), respectively. The ranges of average-NLR were divided into low (2.5–4.9), intermediate (4.9–9.2), and high (9.2–36.6), respectively. The ranges of NLR-nadir were divided into low (0.1–1.5), intermediate (1.5–3.2), and high (3.2–7.9), respectively. The ranges of NLR-maximum were divided into low (4.5–8.7), intermediate (8.7–23.0), and high (23.0–197.5), respectively. The ranges of NLR-post-RT were divided into low (1.6–3.0), intermediate (3.0–6.5), and high (6.5–48.4), respectively.

### Statistical analyses

The primary endpoint was overall survival (OS), which was defined as the time from the start of radiotherapy until death. The secondary endpoints were locoregional progression (LRP) and distant failure (DF). LRP was defined as the progression of disease in the primary tumor, mediastinal, hilar, and supraclavicular lymph nodes. DF was defined as all other patterns of failure. These endpoints were calculated using the Kaplan–Meier method and were compared using the log-rank test. The Cox proportional hazards model was used for the univariate and multivariate analyses. Factors including age, sex, performance status, stage, combined chemotherapy, consolidation chemotherapy, radiotherapy period, NLR-pre-RT, average-NLR, NLR-nadir, NLR-maximum, and NLR-post-RT were analyzed using the univariate and multivariate analyses. In the univariate and multivariate analyses, NLR-pre-RT, average-NLR, NLR-nadir, NLR-maximum, and NLR-post-RT were divided into three groups for clarifying the differences among these factors. The factors with *p* values < 0.10 added to the multivariate analysis. A *p* value of < 0.05 was considered statistically significant. All statistical analyses were performed using EZR (Saitama Medical Center, Jichi Medical University, Saitama, Japan), which is a graphical user interface for R (The R Foundation for Statistical Computing, Vienna, Austria, version 3.6.3) [14]. More precisely, it is a modified version of R commander (2.5–3) designed to incorporate statistical functions frequently used in biostatistics.

## Results

The characteristics of the reviewed 111 patients are listed in Table 1. Seventy-two patients (64.9%) had died at the last follow-up. The median follow-up duration among the survivors was 43.8 months (25.8–107.0 months).

### Overall survival

**Fig. 1:**
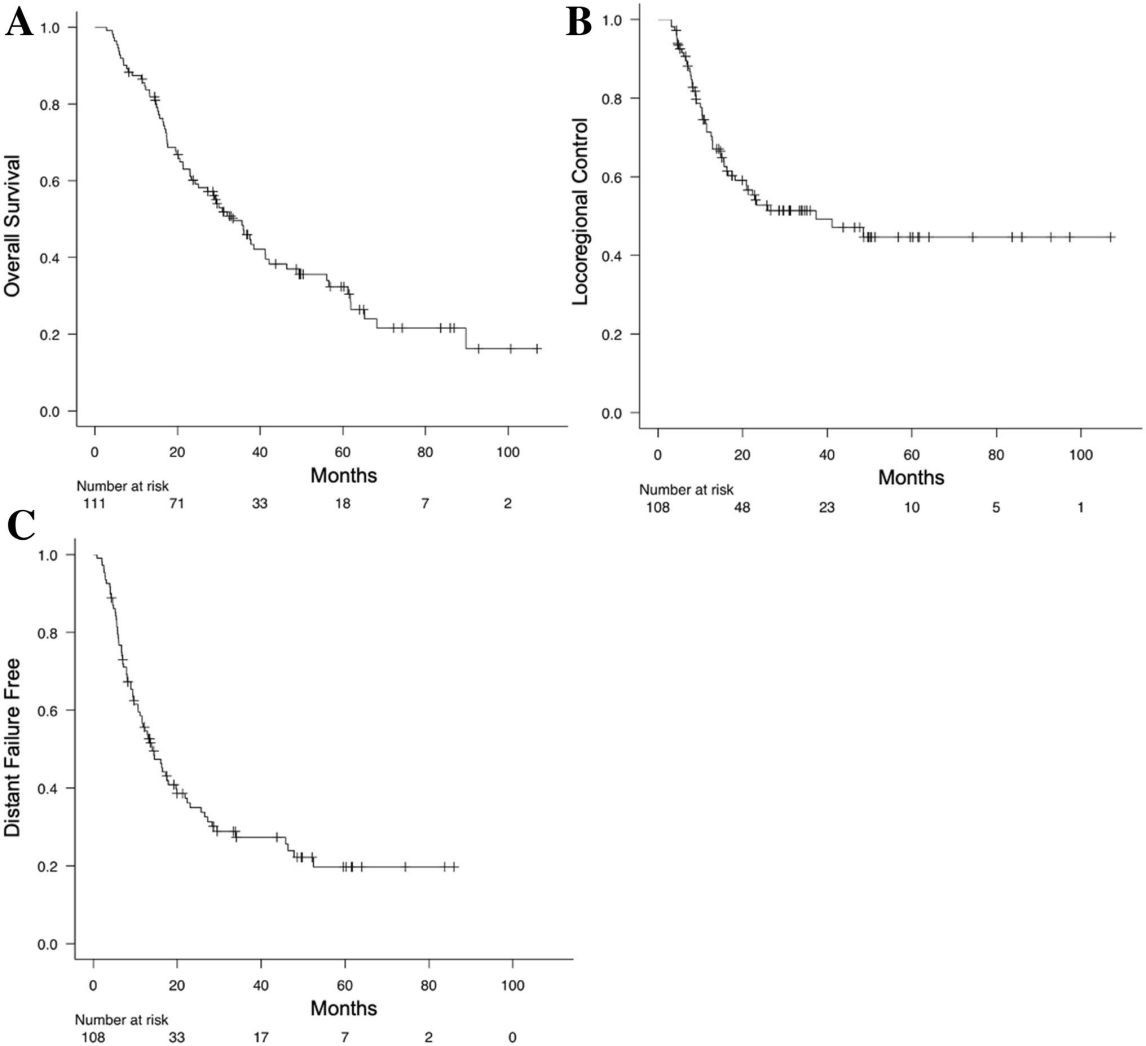
Kaplan–Meier plots. Kaplan–Meier plots for overall survival rate (**a**), locoregional control rate (**b**), and distant failure free rate (**c**) in unresectable stage III non-small cell lung cancer patients

The median OS was 33.4 months, and the 3-year OS rate was 47.2% (Fig. 1a). For the OS, age ≥ 70 years, histology of non-adenocarcinoma, high average-NLR, and high NLR-post-RT were significantly associated with worse OS (*p* = 0.010, *p* = 0.010, *p* = 0.003, and *p* = 0.035, respectively). In the multivariate analysis, high average-NLR and high NLR-post-RT were statistically significant unfavorable factors for the OS (*p* = 0.016 and *p* = 0.028, respectively) (Tables 2 and 3).

**Table 2:** Univariate analysis for overall survival, locoregional progression, and distant failure

		OS		LRP		DF	
		*p* value	HR (95% CI)	*p* value	HR (95% CI)	*P* value	HR (95% CI)
Age (years)	< 70 vs. ≥ 70	0.010	1.91 (1.17–3.10)	0.117	1.59 (0.89–2.85)	0.492	1.18 (0.73–1.91)
Sex	Male vs. female	0.332	0.74 (0.39–1.37)	0.517	0.79 (0.38–1.62)	0.950	0.98 (0.56–1.71)
PS	0, 1 vs. ≥ 2	0.556	1.29 (0.55–2.99)	0.287	1.66 (0.65–4.19)	0.768	1.15 (0.46–2.86)
Histology	ADC vs. non-ADC	0.010	1.87 (1.16–3.00)	0.013	2.07 (1.17–3.69)	0.635	1.12 (0.71–1.75)
Stage	IIIA vs. IIIIB	0.570	1.15 (0.72–1.83)	0.422	0.79 (0.45–1.39)	0.443	1.20 (0.76–1.89)
Combined chemotherapy	Platinum doublet vs. others	0.147	1.80 (0.81–3.97)	0.827	1.12 (0.40–3.13)	0.863	1.08 (0.47–2.49)
Consolidation chemotherapy	Yes vs. no	0.917	1.03 (0.64–1.66)	0.805	0.93 (0.53–1.67)	0.225	1.58 (0.76–3.46)
Treatment period (days)	< 50 vs. ≥ 50	0.577	1.17 (0.67–2.05)	0.408	1.33 (0.68–2.60)	0.859	1.05 (0.60–1.86)
NLR-pre-RT	Intermediate vs. low	0.162	0.66 (0.36–1.18)	0.129	1.10 (0.97–1.24)	0.818	0.94 (0.54–1.63)
	Intermediate vs. high	0.589	1.16 (0.67–2.02)	0.289	1.44 (0.73–2.82)	0.169	1.46 (0.85–2.50)
NLR-nadir	Intermediate vs. low	0.960	0.99 (0.56–1.73)	0.161	0.58 (0.27–1.24)	0.402	1.26 (0.74–2.15)
	Intermediate vs. high	0.515	1.21 (0.68–2.14)	0.707	1.13 (0.59–2.17)	0.370	1.29 (0.74–2.24)
NLR-maximum	Intermediate vs. low	0.610	1.16 (0.66–2.03)	0.125	1.71 (0.86–3.40)	0.642	0.88 (0.50–1.53)
	Intermediate vs. high	0.589	1.17 (0.66–2.07)	0.034	2.07 (1.06–4.07)	0.960	0.99 (0.57–1.71)
NLR-post-RT	Intermediate vs. low	0.572	0.83 (0.45–1.56)	0.204	0.63 (0.30–1.29)	0.990	1.00 (0.57–1.78)
	Intermediate vs. high	0.035	1.87 (1.05–3.34)	0.692	0.86 (0.40–1.82)	0.053	1.75 (0.99–3.08)
Average-NLR	Intermediate vs. low	0.425	0.78 (0.42–1.44)	0.683	1.16 (0.58–2.31)	0.014	0.47 (0.25–0.85)
	Intermediate vs. high	0.003	2.27 (1.31–3.93)	0.018	2.23 (1.15–4.33)	0.185	1.44 (0.84–2.45)

*OS* overall survival, *LRP* locoregional progression, *DF* distant failures, *HR* hazard ratio, *CI* confidence interval, *PS* Eastern Cooperative Oncology Group performance status, *NLR-pre-RT* neutrophil-to-lymphocyte ratio within 2 weeks before radiotherapy, *NLR-nadir* the minimal neutrophil-to-lymphocyte ratio in blood sampling during radiotherapy, *NLR-maximum* the maximal neutrophil-to-lymphocyte ratio in blood sampling during radiotherapy, *NLR-post-RT* neutrophil-to-lymphocyte ratio measured in 3–4 months after radiotherapy, *Average-NLR* the area under the line graph of NLR counts during radiotherapy was divided by days of treatment period, ADC: adenocarcinoma

**Table 3:** Multivariate analysis for overall survival, locoregional progression, and distant failure

			*p* value	HR (95% CI)
OS	Age (years)	< 70 vs. ≥ 70	0.131	1.51 (0.89–2.56)
	Histology	ADC vs. non-ADC	0.119	1.52 (0.90–2.58)
	NLR-post-RT	Intermediate vs. low	0.399	0.76 (0.40–1.44)
		Intermediate vs. high	0.028	1.93 (1.07–3.47)
	Average-NLR	Intermediate vs. low	0.710	0.88 (0.46–1.70)
		Intermediate vs. high	0.016	2.12 (1.15–3.90)
LRP1	Histology	ADC vs. non-ADC	0.013	2.11 (1.17–3.81)
	NLR-maximum	Intermediate vs. low	0.064	1.93 (0.96–3.85)
		Intermediate vs. high	0.063	1.90 (0.97–3.74)
LRP2	Histology	ADC vs. non-ADC	0.038	1.87 (1.04–3.37)
	Average-NLR	Intermediate vs. low	0.723	1.13 (0.57–2.27)
		Intermediate vs. high	0.061	1.91 (0.97–3.77)
DF	NLR-post-RT	Intermediate vs. low	0.656	1.14 (0.64–2.03)
		Intermediate vs. high	0.040	1.83 (1.03–3.25)
	Average-NLR	Intermediate vs. low	0.008	0.42 (0.22–0.80)
		Intermediate vs. high	0.366	1.30 (0.74–2.27)

*OS* overall survival, *LRP* locoregional progression, *LRP1* the multivariate analysis for locoregional progression with histology and NLR-maximum, *LRP2* the multivariate analysis for locoregional progression with histology and average-NLR, *DF* distant failures, *HR* hazard ratio, *CI* confidence interval, *NLR-maximum* the maximal neutrophil-to-lymphocyte ratio in blood sampling during radiotherapy, *NLR-post-RT* neutrophil-to-lymphocyte ratio measured in 3–4 months after radiotherapy, *Average-NLR* the area under the line graph of NLR counts during radiotherapy was divided by days of treatment period, *ADC* adenocarcinoma

**Fig. 2:**
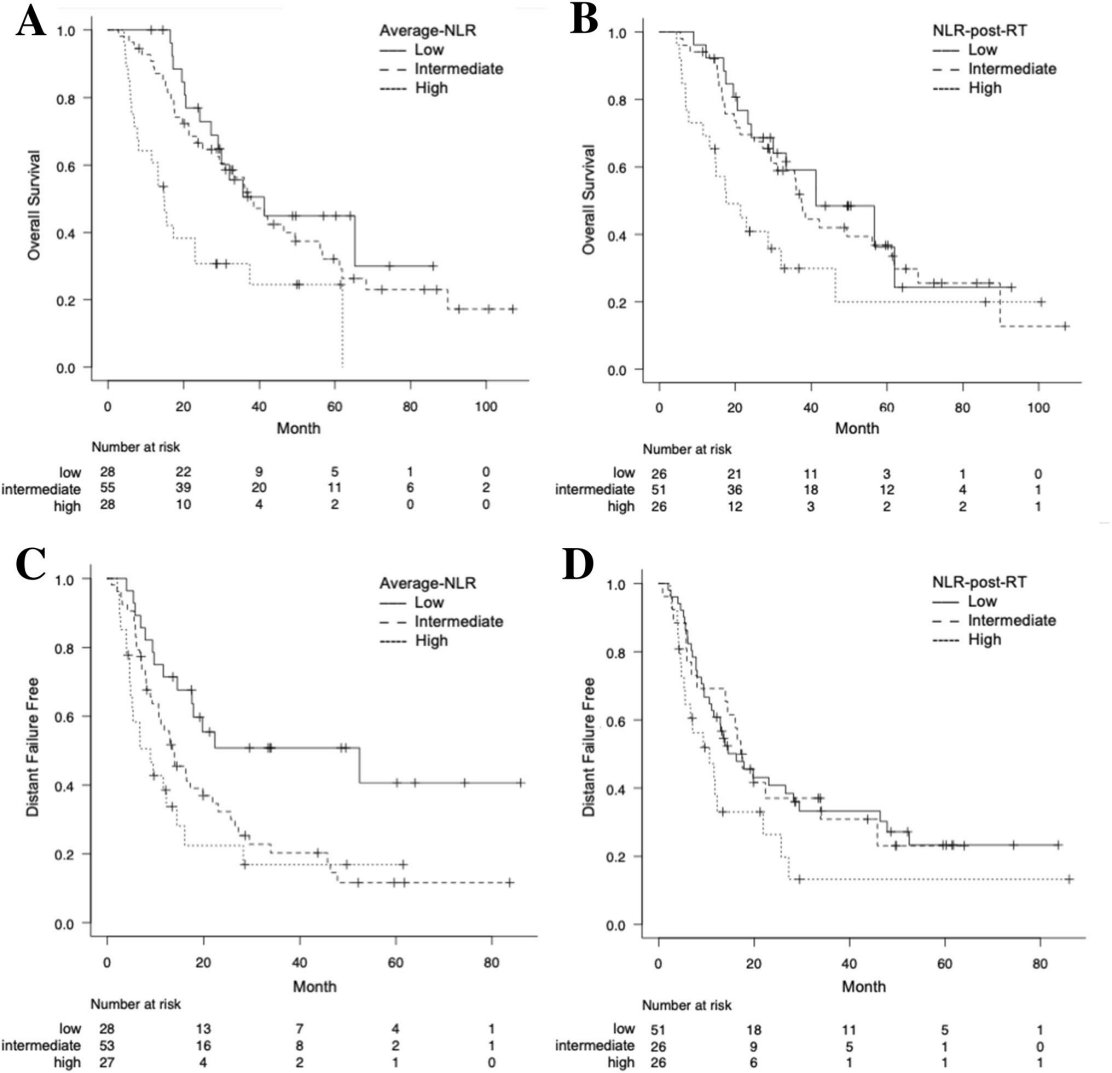
Comparison of overall survival and distant failure in unresectable stage III non-small cell lung cancer patients. For the overall survival, patients were segregated according to low, intermediate, and high in average-neutrophil-to-lymphocyte ratio (NLR) (**a**) and according to low, intermediate, and high in NLR-post-radiotherapy (RT) (**b**). For the distant failure, patients were segregated according to low, intermediate, and high in average-NLR (**c**) and according to low, intermediate, and high in NLR-post-RT (**d**). The Kaplan–Meier curves of the overall survival and distant failure in patients with low, intermediate, and high NLR are represented using solid, dashed, and dotted lines, respectively

The median OS of patients with average-NLR in low, intermediate, and high groups were 41.2, 37.7, and 14.8 months, respectively (*p* = 0.002). The 3-year OS rates in the low, intermediate, and high groups were 50.5, 54.1, and 30.6%, respectively. The median OS of patients with NLR-post-RT in the low, intermediate, and high groups were 41.3, 37.5, and 17.6 months, respectively (*p* = 0.040). The 3-year OS rates in the low, intermediate, and high groups were 59.1, 37.5, and 29.8%, respectively. The Kaplan–Meier curves are shown in Fig. 2a, b.

### Locoregional progression

LRP occurred in 49 patients (44.1%). The median time to LRP was 37.3 months, and the 3-year locoregional control rate was 51.4% (Fig. 1b). In the univariate analysis, histology of non-adenocarcinoma, high NLR-maximum, and high average-NLR were significant factors for LRP (*p* = 0.013, 0.034, and 0.018, respectively). In Spearman’s rank correlation coefficient, we found correlations between average-NLR and NLR-maximum (*r* = 0.852, *p* = 0), and we analyzed NLR-maximum and average-NLR in separate multivariable models. In the multivariate analysis, adenocarcinoma was a significant factor for LRP (*p* = 0.013 in model 1, and *p* = 0.038 in model 2) (Tables 2 and 3). The median time to LRP in the adenocarcinoma and non-adenocarcinoma cohorts was not reach and 18.2 months, respectively. The 3-year LRP free rates in the adenocarcinoma and non-adenocarcinoma cohorts were 61.1 and 40.9%, respectively.

### Distant failure

DF occurred in 90 patients (81.1%). The median time to DF was 14.0 months, and the 3-year DF free rate was 27.3% (Fig. 1c). In the univariate analysis, low average-NLR was a significant favorable factor for DF (*p* = 0.014). In the multivariate analysis, low average-NLR was a significant favorable factor for DF (*p* = 0.008), and high NLR-post-RT was a significant unfavorable factor for DF (*p* = 0.040) (Tables 2 and 3).

The median time to DF of patients with low, intermediate, and high average-NLR were 52.5, 13.5, and 8.9 months, respectively (*p* = 0.004). The 3-year DF free rates in the low, intermediate, and high groups were 50.8, 20.3, and 16.8%, respectively. The median time to DF of patients with low, intermediate, and high NLR-post-RT were 17.4, 16.3, and 10.7 months, respectively (*p* = 0.114). The 3-year DF free rates in the low, intermediate, and high groups were 33.2, 30.9, and 13.2%, respectively. The Kaplan–Meier curves are shown in Fig. 2c, d.

## Discussion

We evaluated the clinical significance of NLR-related factors, including average-NLR, NLR-pre-RT, NLR-post-RT, NLR-nadir, and NLR-maximum in patients who received CRT for unresectable stage III NSCLC. Among these metrics, average-NLR and NLR-post-RT were associated with the OS and DF. NLR-pre-RT and NLR-nadir were not statistically significant for treatment outcomes. Low average-NLR was associated with low incidence of DF, while high average-NLR was associated with poor OS and a tendency of unfavorable LRP. Although appropriate cut-off points of average-NLR for OS and DF differed, average-NLR was seemed to be a good prognostic factor for OS and DF.

Although both average-NLR and NLR-post-RT were significant factors for the OS, average-NLR seemed to have some merits than NLR-post-RT. First, the prediction of treatment outcomes could be performed earlier when average-NLR was used. Average-NLR could be immediately calculated at the end of radiotherapy, while NLR-post-RT could be obtained a few months after the completion of radiotherapy. Earlier prediction leads to earlier decision making for adjuvant therapy and follow-up interval. Second, average-NLR might have a greater impact on DF compared to the NLR-post-RT. The differences in the DF free rates after 3 years were more apparent when stratified by aggregate-NLR compared to when stratified by NLR-post-RT. Average-NLR seemed to be more useful in predicting the development of long-term DF. Thus, we believed that average-NLR has two advantages over NLR-post-RT: immediate availability at the end of radiotherapy and more precise prediction for long-term risk of DF.

According to previous reports, NLR before CRT [9, 10], NLR after CRT [11, 12], NLR during CRT [12], and NLR-nadir [11] were useful prognostic factors in definitive radiotherapy with or without chemotherapy for NSCLC, and the results varied depending on the study. Scilla et al. assessed 276 patients with stage III NSCLC treated with combined modality therapy based on CRT and found that there was a significant association between the pretreatment NLR and OS [9]. Kang et al. reviewed 163 patients with stage III NSCLC who received definitive CRT with or without surgery. They found that a combination of high pretreatment NLR and low lymphocyte nadir during CRT was significantly associated with the OS [10]. These results were not consistent with our findings. Scilla et al. reported that there were associations between lower pretreatment NLR and the addition of surgery [9], and approximately 30% of patients underwent CRT and surgery. Kang et al. also included 41% of patients underwent combination therapy between CRT and surgery in their study [10]. These may be one of the possible explanations that their results were different from ours because patients who underwent surgery were excluded from our study. Conteras et al. reviewed 400 patients with stage II–III NSCLC and showed that the NLR at 4 months after the start of radiotherapy was a prognostic factor for OS and DF [11]. In addition, their results were consistent with ours in that NLR-pre-RT and NLR-nadir were not significant prognostic factors for the OS. Thor et al. examined the relationship between the change in NLR from the start of radiotherapy to several months after the radiotherapy and treatment outcomes in patients who received CRT for stage III NSCLC [12]. A larger decrease in NLR at 4 months after the start of radiotherapy was associated with worse OS. In contrast, change rates in NLR during radiotherapy (from 12th day to 2 months after the start of radiotherapy) were not associated with the OS. Their results were only based on the comparison of the NLR at 12 days and several months after the start of radiotherapy and did not reflect the details of NLR transition throughout the course of radiotherapy. Different from change rates of NLR between before and after radiotherapy, average-NLR has potential to more accurately reflect inflammatory and immune response throughout radiotherapy.

In our study, the DF free rate was higher in patients with low average-NLR than in patients with intermediate and high average-NLR, and this difference did not decrease after 3 years. This suggests that average-NLR, which reflects the inflammatory and immune response throughout radiotherapy have an impact on tumor progression, has the potential to contribute to disease control out of irradiation fields. Several studies have reported that lymphopenia and higher NLR were associated with worse survival in NSCLC patients who received immunotherapy with or without radiotherapy [15–18]. Considering the results of these studies, lymphopenia and higher NLR may reflect deterioration of the immune response. Moreover, Chen et al. reported that in patients who received combined immunotherapy and radiotherapy, a higher absolute lymphocyte count was associated with not only better OS but also higher abscopal response rate [19]. The results of this study may support our results that high average-NLR was associated with a high DF rate. Although further studies are needed, deterioration of the immune response associated with lymphopenia and higher NLR contributes to DF rather than LRP in patients who received CRT for unresectable stage III NSCLC. Average-NLR also might enable to predict the effectiveness of consolidation immunotherapy immediately after CRT.

Our study has several limitations, including its retrospective nature, small sample size, various combined chemotherapies, and consolidation chemotherapy. In addition, NLR potentially has the concerns of objectivity and reproducibility due to the effects of transient infection and treatment. Although blood samplings were performed every week in principle, blood sampling timing slightly varied patient by patient. The timing and number of blood sampling slightly affected the value of average-NLR. However, at least seven times blood sampling data were used to calculate average-NLR in approximately 95% of patients. In addition, average-NLR are thought to reduce the influence of timing and number of blood sampling by means of taking average rather than NLR at certain time point. Therefore, it seemed that the effects of blood samplings timing and events on average-NLR were reduced. Furthermore, average-NLR is certainly more complicated to calculate than a simple average of NLR throughout radiotherapy, but it has the advantages of being less sensitive to blood sampling intervals and less likely to overestimate changes in NLR due to infection or treatment. Nevertheless, in conclusion, our results suggest that average-NLR is clinically helpful for the prediction of OS and DF immediately after CRT in unresectable stage III NSCLC patients who underwent concurrent CRT. The novelty of our study was to devise an average-NLR and show its potential as a prognostic factor. However, further studies are needed to better understand the impact of inflammatory and immune responses on the survival and tumor control during the entire CRT course.
